# Evaluation of long-term efficacy and safety of dienogest in patients with chronic cyclic pelvic pain associated with endometriosis

**DOI:** 10.1007/s00404-023-07271-7

**Published:** 2023-11-29

**Authors:** Antonio Maiorana, Marianna Maranto, Vincenzo Restivo, Daniele Lo Gerfo, Gabriella Minneci, Antonella Mercurio, Domenico Incandela

**Affiliations:** 1 HCU Obstetrics and Gynecology, ARNAS Civico Di Cristina-Benfratelli Hospital, Palermo, Italy; 2 Department of Medicine, University Kore of Enna, Enna, Italy; 3 G.F. Ingrassia Hospital, Palermo, Italy

**Keywords:** Endometrioma, Dysmenorrhea, Dyspareunia, Dyschezia, Cyclic pelvic pain

## Abstract

**Purpose:**

To evaluate the efficacy and long-term safety (up to 108 months) of treatment with Dienogest in patients with endometriosis.

**Methods:**

Patients with chronic pelvic pain endometriosis-related were enrolled in this observational study from June 2012 to July 2021. The patients enrolled took Dienogest 2 mg as a single daily administration. Group B of long-term therapy patients (over 15 months) were compared with group A of short-term therapy patients (0–15 months). The effects of the drug on pain variation were assessed using the VAS scale and endometriomas dimensions through ultrasonographic evaluation. Furthermore, has been valuated the appearance of side effects and the effect of the drug on bone metabolism by performing MOC every 24 months in group B.

**Results:**

157 patients were enrolled. The mean size of the major endometrioma progressively decreased from 33.2 mm (29.4–36.9) at T0 to 7 mm (0–15.8) after 108 months of treatment. We found a significant improvement in dysmenorrhea, dyspareunia, dyschezia and non-cyclic pelvic pain. As for the side effects, both groups complained menstrual alterations present in 22.9%. In 27.6% of group B, osteopenia was found. Group B had a higher percentage statistically significant of side effects such as headaches, weight gain and libido reduction compared to group A. 2

**Conclusion:**

Long-term therapy with Dienogest has proven effective in controlling the symptoms of the disease and reducing the size of endometriomas, with an increase in the positive effects related to the duration of the intake and in the absence of serious adverse events. Study approved by the "Palermo 2" Ethics Committee on July 2, 2012 No. 16.

## What does this study add to the clinical work

**Table:** 

Long-term therapy with Dienogest 2 mg has effective in controlling the symptoms of the disease and reducing the size of endometriomas, with an increase in the positive effects progressively related to the duration of the intake.
Furthermore this study demonstrates the long-term safety of dienogest up to a treatment period of 108 months, in the absence of serious adverse events.

## Introduction

Endometriosis is a pathological condition characterized by the dissemination and growth of endometrial tissue in abnormal locations, with stromal, epithelial and glandular characteristics similar to those of the normal endometrium. The ectopic endometrium is affected, like the normal uterine mucosa, by stimuli from ovarian hormones, especially estrogens, and therefore, assumes proliferative and functional attitudes similar to those occurring in the normal endometrium.

It is estimated that 10–15% of women of reproductive age suffer from pelvic endometriosis. This disease has been classified by AFS into four stages based on the severity, quantity, location, depth and size of the endometriotic foci. Endometriosis has been shown to be an estrogen-dependent pathology and progesterone-dependent, in fact, it develops in women of reproductive age and regresses after menopause or ovariectomy, suggesting that the formation and growth of ectopic implants depends on ovarian steroids, similar to eutopic endometrium.

While a significant number of women with this condition can be asymptomatic, others often exhibit severe dysmenorrhea, chronic pelvic pain (CPP), dysfunctional uterine hemorrhage, infertility, dyspareunia, dyschezia, urinary tract symptoms and gastrointestinal symptoms [1–4].

Diagnosis of endometriosis is first suspected on the basis of medical history, symptoms and signs, in association with physical examination and imaging techniques. There are no pathognomonic features or biomarkers necessary and sufficient to define endometriosis [5].

Transvaginal ultrasound is useful for identifying or excluding the presence of endometriosis-related lesions but the definitive diagnosis is demonstrated by histological examination of the samples collected during laparoscopy [6, 7]. Even if the current shared diagnostic paradigm requires as a gold standard laparoscopy with histological endometriosis verification, many support the treatment of symptoms before obtaining a definitive surgical diagnosis [8]. In particular, the 2017 guidelines of the National Institute for Health and Care Excellence propose a philosophical change, endorsing empirical therapy before laparoscopy in diagnostic and therapeutic algorithm.

The main management options in endometriosis include surgery, hormone therapy, or a combination of these two approaches. Excision of endometrial lesions provides rapid symptom relief, but lesion recurrence rates are high, estimated at 40–50% at 5 years [9, 10]. For these women, postoperative hormone therapy can reduce recurrence of lesions and prolong the period without pain [11, 12]. Hormonal therapy is also widely used as a first-line therapy in symptomatic women and in cases where surgery is not recommended or is rejected by the patient [11, 13–16].

Commonly used hormonal therapies include GnRH agonists, estroprogestins and progestins. Each hormone therapy aims to reduce the size of endometriotic foci and the associated symptoms by exploiting the estrogen reactivity characteristic of endometrial lesions [11].

Progestins are a viable option to inhibit the proliferation of estrogen-induced lesions and reduce pain associated with endometriosis. Among progestins approved for the treatment of endometriosis, Dienogest has demonstrated, in the various studies, a powerful progestogenic action, a moderate estrogen suppressive effect, anti-inflammatory, antiproliferative and antiangiogenic properties that effectively reduce the growth of endometriosic lesions [17–20]. Clinical studies in Europe and Asia have shown that Dienogest, administered at a daily dose of 2 mg, provided pain relief in endometriosis significantly higher than placebo and equivalent to GnRH as, with fewer hypoestrogenic effects than the latter [21–23]. Further studies have shown that Dienogest is more effective than norethindrone acetate, another progestin used to treat endometriosis, with less risk of adverse effects [24, 25]. Clinical studies lasting up to 15 months have shown that Dienogest 2 mg provides a continuous reduction of injuries and pain relief, associated with improvements in the quality of life of patients [26–28]. Prolonged reductions in symptoms were also described in monocentric cohort studies lasting 6–12 months [29–31], while long-term cohort studies (up to 60 months) reported significant reductions in post-surgical relapses with Dienogest 2 mg compared to patients who had not undergone any medical treatment [32, 33]. Dienogest 2 mg has a favorable safety profile, characterized by mild hypoestrogenic effects, minimal effect on bone mineral density in adult women and low withdrawal rates of treatment [22, 23, 26, 27]. Instead, published studies on the efficacy and safety of Dienogest for treatment periods of more than 15 months are currently limited.

This study describes the experience of our center, with Dienogest 2 mg, in women with endometriosis, for a period of treatment up to 108 months, furthermore compares the group of patients who practiced long-term therapy with those who practiced short-term therapy.

## Materials and methods

We conducted an observational study on 157 patients suffering from CPP pain who have obtained a clinical or a surgical diagnosis of endometriosis. Inclusion and exclusion criteria are described in Table 1.

**Table 1:** Inclusion/exclusion criteria

*The inclusion criteria were*
Age over 18 and under or equal to 54
Surgical diagnosis of endometriosis and/or adenomyosis
Clinical diagnosis of endometriosis and/or adenomyosis (confirmed by clinical history, bimanual pelvic examination, TV and/or TR ultrasound)
Clinical indication for medical therapy (chronic cyclic pelvic pain, prevention of relapses)
No need for contraception
*The exclusion criteria were*
Age under 18 and over 54
Suspicion of neoplasm
Immediate reproductive desire
Contraindication to progestin therapy
Simultaneous use of NSAIDs
Recourse to a pain therapy center

Patients were recruited from June 2012 to July 2021 at the Center for the diagnosis and treatment of endometriosis of the ARNAS Civic Hospital in Palermo. All patients were adequately informed about the conduct of the study. The patients enrolled in this study were administered Dienogest 2 mg as a single daily administration, in the evening and always at the same time.

Although Dienogest has been authorized to market in Italy since February 2013, some patients had started taking it earlier, through spontaneous purchase on the European market.

All enrolled patients were followed clinically in a Center of Excellence for the diagnosis and treatment of endometriosis with the presence of an expert gynecologist, with a dedicated psychologist and nurse and in collaboration with a radiologist, gastroenterologist, abdominal surgeon, urologist and thoracic surgeon, all with specific experience and belonging to a multidisciplinary hospital group dedicated to endometriosis.

The effects of Dienogest 2 mg on long-term pain variation were assessed using the VAS scale and endometriosis lesion dimensions through ultrasonographic pelvic abdomen evaluation. In addition, has been valuated the appearance of side effects (head-ache, spotting, reduced libido and weight gain) and the effect of the drug on bone metabolism by performing evaluation of bone mineral density determined by dual-energy X-ray absorptiometry (DXA) every 24 months, comparing them with the same values described in the literature for unaffected women, of the same age and characteristics.

Group B of long-term therapy patients (more than 15 months) were compared with group A of short-term therapy patients (0–15 months) comparing the response to pain and the appearance of adverse or side effects. The treatment has been defined, in the present study, as “short term” in patients who have performed therapy up to 15 months and “long term” in patients who have performed therapy for more than 15 months.

Patients were clinically evaluated at 0, 6, 12, months and then every 12 months. All women underwent standardized medical history collection, gynecological examination and transvaginal/transrectal/transabdominal ultrasound. The examination consisted in the inspection of the vagina and cervix using the speculum, in the systematic palpation of the abdomen and pelvis, adding the digital-anorectal exploration. Patients were subjected to transvaginal and/or transrectal ultrasound by the same operators who performed the examination, in order to obtain a study of the pelvic organs, the possible presence of ovarian lesions, signs of pelvic distortion, endometriotic lesions of the anterior, lateral and posterior pelvic compartments. In all patients, during each ultrasound examination, the research of possible hydronephrosis was carried out in order to evaluate a ureteral involvement.

Only the volume of the larger endometrioma and its size changes during the duration of the therapy were included in this study.

The pain intensity was assessed using the visual analog scale of pain (VAS). The reference symptoms evaluated in this study were: dysmenorrhea, dyspareunia, dyschezia and non-menstrual pain [34].

VAS has been shown to be sensitive to changes in the patient’s endometriosis-related pain experience. It is fast to use and relatively easy to understand for most patients. Avoids imprecise use of words to describe pain and allows meaningful comparison of measurements over time [35].

The VAS score was recorded at baseline T0 and after 12 month T1, 24 T2, 36 T3, 48 T4, 60 T5, 72 T6, and 84 T7 months.

In addition to the evaluation of pain, at each clinical check, were evaluated all the side effects reported by the patient and in particular those that in the literature are more statistically correlated to the therapy practiced as the appearance of genital bleeding, headache, weight gain and libido reduction.

Regarding the statistical study categorical variables as percentage, quantitative variables were summarized as mean (95% confidence intervals) and median (interquartile distance). Fisher’s exact-test for contingency tables, *z* test and non-parametric Mann–Whitney *U* test were used when appropriate. A multivariate logistic analysis was used to assess the relationship between variables and clinical diagnosis. Variables were chosen according to the Hosmer–Lemeshow methodology. After univariate analysis, only variables with a *p* < 0.20 were included in the final model; then, through a back-ward process, variables were excluded until a significance level of *p* < 0.20 was reached for each variable. The application of Hosmer–Lemeshow test is a measure of how well the model fits the data without any choice of variables by researcher to put into the multivariate model. A two-tailed *p* < 0.05 was considered statistically significant. Stata Statistical Software2016, Release14 (Stata-Corp, College-Station, TX-USA) was used for database management and all the analyses.

## Results

Out of 195 patients with clinical and surgical diagnosis of endometriosis, who met the inclusion criteria, 157 were enlisted in this study after obtaining consent. The average age of the patients was 37 years (19–54). The median age of the menarche was 12 years (11–13). Of these patients, the majority, 56% had received a surgical diagnosis and a histological confirmation of the disease, while 44% an empirical diagnosis of endometriosis based on symptomatology and ultrasound aspects. Most of the patients (64%) who had obtained a surgical diagnosis were classified as III–IV stage according to the classification of the American Fertility Society revised. Endometriomas were present on the right in 68.9% and on the left in 64.1%. They were bilateral in 27.2%. Endometriosis of the vaginal rectum or rectum-sigma was present in 30.6%. 38.9% reported infertility history (Table 2).

**Table 2:** Characteristics of the study population

*N*	157
Age^a^	37 (19–54)
Menarche^b^	12 (11–13)
Surgical diagnosis (%)	56.0
Clinical diagnosis (%)	44.0
Months of treatment^b^	36 (7–72)
Endometriosis on the right (%)	68.9
Endometriosis on the left (%)	64.1
Bilateral endometriosis (%)	27.2
SRV/RS (%)	30.6
Sterility (%)	38.9
Number of surgeries^b^	1 (0–1)
Staging^b^	4 (4–4)

^a^Data are reported as mean (95% confidence interval)

^b^Data are reported as a median (Q1–Q3)

In our study, 56 patients have been treated for less than 15 months, 27 patients between 15 and 24 months, 7 patients for 24 months, 7 patients for 36 months, 5 patients for 48 months, 12 patients for 60 months, 20 patients for 72 months, 15 patients for 84 months, 7 for 96 months and 1 patient for 108 months. We found a significant reduction in the volume of the larger endometrioma (Table 3).

**Table 3:** Mean change in the volume of the largest endometrioma over time, in patients treated with Dienoget 2 mg

Size of the endometrioma (mm) at T0^a^	33.2 (29.4 to 36.9)
Size of the endometrioma (mm) at T1^a^	24.6 (20.9 to 28.4)
Variation from T0 to T1 of size the endometrioma (mm)^a^	− 8.7 (− 11.5 to − 5.8)
Variation from T0 to T1 of size the endometrioma (%)^a^	− 25.8 (− 34.0 to − 17.6)
Size of the endometrioma (mm) at T2^a^	25.3 (19.7 to 30.9)
Size of the endometrioma (mm) at T3^a^	22.5 (17.3 to 27.7)
Size of the endometrioma (mm) at T4^a^	16.2 (12.3 to 20.1)
Size of the endometrioma (mm) at T5^a^	14.0 (3.2 to 24.8)
Size of the endometrioma (mm) at T6^a^	10.3 (2.7 to 17.9)
Size of the endometrioma (mm) at T7^a^	7.0 (0 to 15.8)

^a^Data are reported as mean (95% confidence interval)

At 12 months, there was an average reduction in mm of 8.7, equal to 25.8% (CI 34–17.6). At 24 months, in patients who had continued long-term therapy, the mean endometrioma size was 25.3 mm (19.7–30.9), 36 months 22.5 mm (17.3–27.7), 48 months 16.2 mm (12.3–20.1), 60 months 14 mm (3.2–24.8), 72 months 10.3 mm (2.7–17.9), 84 months 7 mm (0–15.8).

We found a significant improvement in endometriosis-related symptoms with reduction of major symptoms such as dysmenorrhea, dyspareunia, dyschezia and pelvic pain not related to menstruation.

**Fig. 1:**
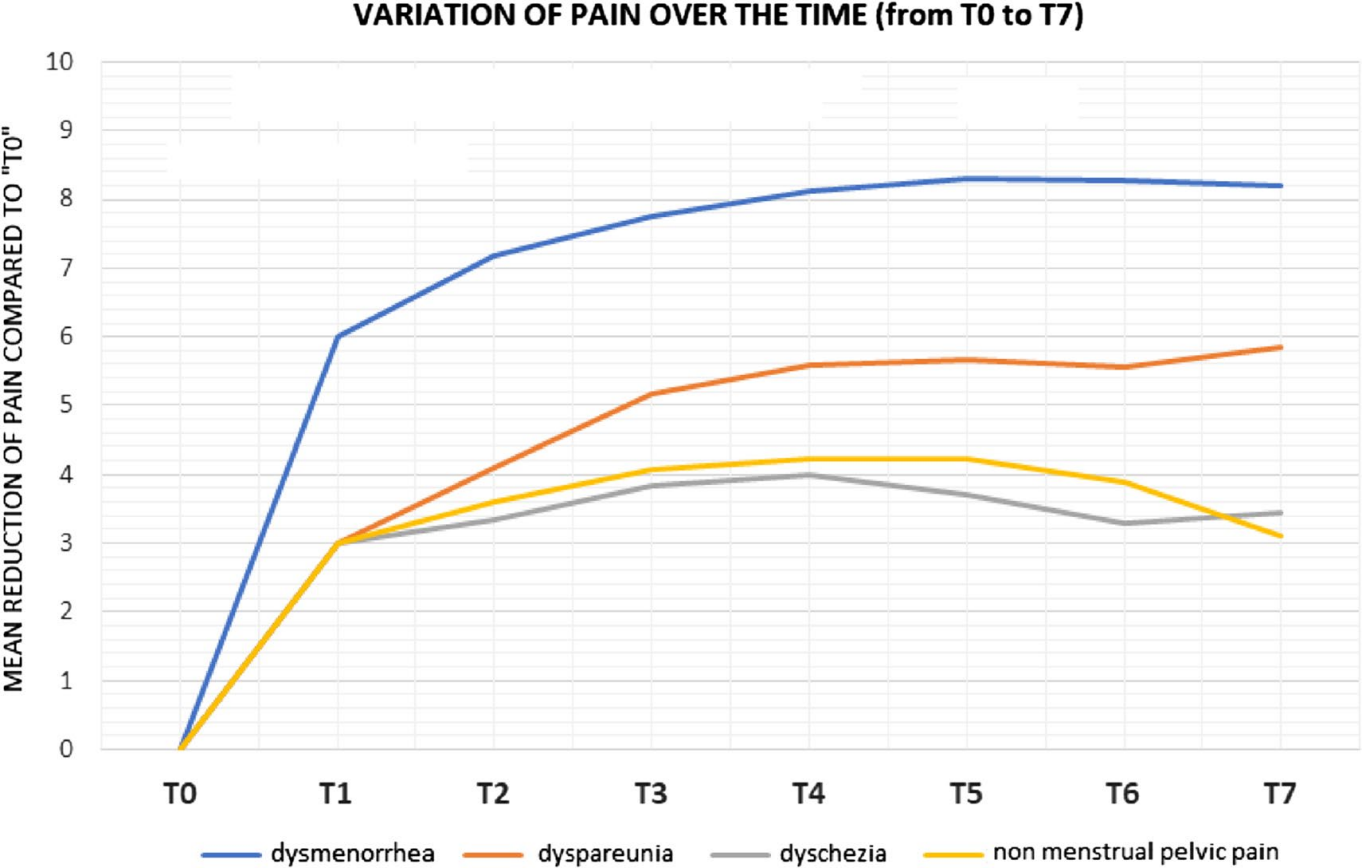
Variation of pain over time (from T0 to T7) in patients treated with Dienogest 2 mg

The graph in Fig. 1 shows the average pain reduction between T0 and T7. It should be noted that dysmenorrhea has had a more significant reduction than the other symptoms, which, however, show a positive improvement trend.

At T0, the mean value for dysmenorrhea was 8, at T7 was 0.1. For dyspareunia at T0 was 5.9, at T7 0.65. T0 dyschezia was 3.8, T7 was 0.45. T0-related pelvic pain was 4.8, T7 was 0.

**Fig. 2:**
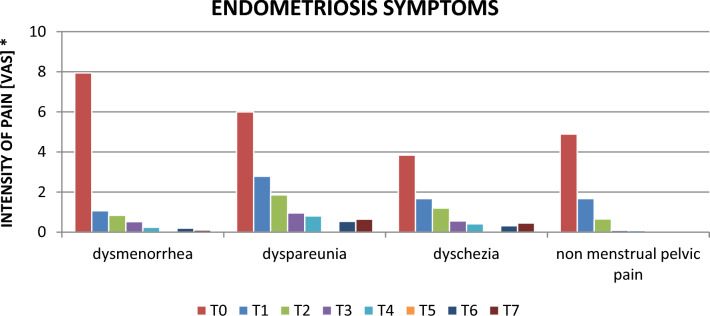
The Figure makes the pain reduction over time clearer

As the graph in Fig. 2 shows, there has been a clear reduction in the average intensity of dysmenorrhea between T0 to T1, while for other symptoms, this reduction has been more gradual. In particular, with regard to dyspareunia and dyschezia, the patients who suffered from these symptoms declared a clear improvement in the symptomatology, complaining of a slight pain (referred to as discomfort) during sexual intercourse and during defecation. With regard to the side effects of Dienogest, short-term therapy patients, as well as those undergoing long-term treatment complained, only in the first months of treatment, menstrual alterations (in particular spotting) present in 22.9% and headaches, present in 17.2%. Weight gain and libido reduction were present in 17.8% and 32.7%, respectively, in the total of Group A and Group B patients (Table 4).

**Table 4:** Side effects drug related in the study population

Spotting (%)	22.9
Headache (%)	17.2
Weight gain (%)	17.8
Reduced libido (%)	32.7
Osteopenia (%)	27.6

Patients who had been receiving treatment for more than 15 months were advised to perform bone densitometry every 24 months. In 27.6% of these patients, who had been receiving treatment for more than 15 months, osteopenia was found.

A comparison was made between the patients who had stopped therapy with Dienogest before 15 months and those who had been treated for more than 15 months (Table 5). Patients who had done short-term therapy were younger than those who had done long-term therapy (*p* value < 0.0001). The latter had obtained a higher percentage surgical diagnosis than those with short-term therapy (*p* 0.0320) and had a higher percentage bilateral endometriosis (*p* 0.0006) and endometriosis of SRV/RS (*p* 0.0010). In relation to the long-term tolerability of the drug, long-term therapy patients had a higher percentage of side effects such as headaches (*p* 0.0008) weight gain (*p* 0.0024) and libido reduction (*p* < 0.0001) compared to patients who had stopped therapy before 15 months.

**Table 5:** Table of comparison between patients who have done short-term therapy with those who have done long-term therapy

Variables	Short-term therapy (*n* = 56)	Long-term therapy (*n* = 102)	*p*
Age^a^	32.5 (30.5–34.5)	40.0 (38.5–41.5)	** < 0.0001**
Menarche^a^	12.0 (11.6–12.4)	11.9 (11.7–12.2)	0.6837
Surgical diagnosis (%)	44.6	62.4	**0.0320**
Endometriosis on the right (%)	69.2	68.7	0.9633
Endometriosis on the left (%)	53.8	67.5	0.2072
Bilateral endometriosis (%)	10.7	36.3	**0.0006**
SRV/RS (%)	14.3	39.6	**0.0010**
Spotting (%)	19.6	24.7	0.4657
Headache (%)	3.6	24.7	**0.0008**
Weight gain (%)	5.3	24.7	**0.0024**
Reduced libido (%)	10.7	45.0	** < 0.0001**
Number of surgeries^b^	0 (0–1)	1 (0–1)	**0.0035**
Size of the endometrioma (mm) at T0^a^	33.2 (26.5–39.9)	33.2 (28.5–37.8)	0.7884
Size of the endometrioma (mm) at T1^a^	22.2 (15.8–28.5)	26.2 (21.5–31.0)	0.2306
Variation from T0 to T1 of size the endometrioma (mm)^a^	− 11.2 (− 17.3 to − 5.1)	− 6.9 (− 9.6 to − 4.3)	0.1275
Variation from T0 to T1 of size the endometrioma (%)*	− 31.3 (− 47.3 to − 15.3)	− 22.1 (− 31.1 to − 13.1)	0.0817

Statistically significant *p* values are highlighted in bold

## Discussion

Long-term endometriosis treatment is related to the need to control over time CPP and the incidence of relapses. Some studies have shown that in many patients the withdrawal of the drug was prescribed by their gynecologist only for lack of reassuring clinical studies, despite the effectiveness.

As for pain, a progressive reduction of dysmenorrhea, dyspareunia, dyschezia, non-menstrual pain has been observed, to the satisfaction of patients and the opportunity to avoid future surgery, as demonstrated by several previous studies [27, 36, 37]. With regard to ultrasonographic aspects, the volume of the largest endometrioma was used as a reference to assess the effectiveness of the therapy. In the various checks, there has been a reduction in its volume and in some cases its disappearance. The data relating to the volumetric variation of deep infiltrating endometriotic nodules was not included in this study because a non-homogeneous collection of data with regard to this specific aspect. Many other studies have shown that Dienogest was effective in reducing the size of endometrioma [38–40].

The side effects associated with the therapy occurred generically in very low percentages and few patients decided to discontinue the therapy in relation to side effects. Some patients have stopped therapy for fear of unknown long-term effects while a higher percentage of patients have stopped the drug for reproductive need. Menstrual disorders, especially spotting, occurred in a minority of patients, mainly in the first months of treatment and in accordance with literature data. Symptoms such as headache, weight gain and above all a reduction in libido have occurred in variable percentages, but still low and which tended to occur with greater frequency in group B patients. These patients were also asked to perform bone densitometry every 24 months. In our sample, there were no cases of osteoporosis, while osteopenia was found in less than a third of patients who were doing long-term therapy and this data does not differ substantially from that observed in the general population as conducted by the study ESOPO (Epidemiological Study On the Prevalence of Osteoporosis), performed in 2000.

Comparing the two group, in group B, the average age was about 10 years higher than the group A. Long-term therapy patients were generally not eager for offspring, had already undergone one or more interventions and tried to minimize the risk of re intervention. This group has shown greater adherence to therapy. Short-term therapy patients were usually younger with a milder symptomatology and in many cases stopped therapy to look for a pregnancy or because they were looking for a contraceptive method.

We found no statistically significant differences between the group of patients with clinical diagnosis and those with surgical diagnosis. In fact, both cohorts of patients have shown, regardless of age and severity of symptoms, response to treatment not substantially different. This supports the choice to use clinical diagnosis as the only way to manage the clinical path of the patient avoiding the use of surgery when it is carried out only for diagnostic confirmation. In support of our thesis, the study of Anjeza Xholli et al. suggests that reducing the need for surgery and its potential damage to the ovaries, early diagnosis and appropriate medical treatment can help preserve the ovarian reserve and the future fertility of women [40]. With regard to the treatment of patients already undergoing one or more surgeries, it should be noted that the administration of Dienogest, in addition to having a positive effect on pain, reduces the risk of recurrence.

As demonstrated by a recent meta-analysis by Zakhari et al. of ten studies conducted in Japan out of a total of 2030 patients concluded that patients receiving Dienogest after endometriosis conservative surgery have a low rate of recurrence of the disease compared to their untreated counterparts [41].

The incidence of ovarian neoplasia was absent in both groups, testifying to the high sensitivity and specificity of ultrasound in ovarian endometriosis diagnostics.

In conclusion, long-term therapy with Dienogest 2 mg has proven effective in controlling the symptoms of the disease and reducing the size of endometriomas, with an increase in the positive effects progressively related to the duration of the intake and in the absence of serious adverse events. Patients who participated in this study were willing to tolerate the side effects of the drug for the benefit of substantial pain relief, showing overall an improvement in quality of life. A limitation of the study is given the small sample size, especially for the maximum duration of observation. No studies have been found in literature of such long duration on the effects of Dienogest. Further comparison studies on its long-term efficacy and safety will be needed between patients who practice long-term medical therapy and those who do not. However, this study has proved to be valid in order to extend the period of treatment, ensuring a control of symptoms and an adequate quality of life, in view of an endometriosis management focused on the person rather than on the disease.

## Data Availability

Data will be provided after a motivated request.

## References

[1] Mehedintu C, Plotogea MN, Ionescu S, Antonovici M (2014). Endometriosis still a challenge. J Med Life.

[2] Barcena de Arellano ML, Gericke J, Reichelt U, Okuducu AF, Ebert AD, Chiantera V, Schneider A, Mechsner S (2011). Immunohistochemical characterization of endometriosis-associated smooth muscle cells in humanperitoneal endometriotic lesions. Hum Reprod.

[3] Mounsey A, Wilgus A, Slawson D (2018). Diagnosis and management of endometriosis. Am Fam Phys.

[4] Judge LC, Kao LC (2004). Endometriosis. Lancet.

[5] Johnson NP, Hummelshoj L, Adamson GD, Keckstein J, Taylor HS, Abrao MS, Bush D, Kiesel L, Tamimi R, Sharpe-Timms KL, Rombauts L, Giudice LC, World Endometriosis Society Sao Paulo Consortium (2017). World Endometriosis Society consensus on the classification of endometriosis. Hum Reprod.

[6] Kennedy S (2006). Should a diagnosis of endometriosis be sought in all symptomatic women?. Fertil Steril.

[7] Wykes CB, Clark TJ, Khan KS (2004). Accuracy of laparoscopy in the diagnosis of endometriosis: a systematic quantitative review. BJOG.

[8] Dunselman GA, Vermeulen N, Becker C (2014). ESHRE guideline: management of women with endometriosis. Hum Reprod.

[9] Römer T (2018). Long-term treatment of endometriosis with dienogest: retrospective analysis of efficacy and safety in clinical practice. Arch Gynecol Obstet.

[10] Guo SW (2009). Recurrence of endometriosis and its control. Hum Reprod Update.

[11] Dunselman GA, Vermeulen N, Becker C, Calhaz-Jorge C, D'Hooghe T, De Bie B, Heikinheimo O, Horne AW, Kiesel L, Nap A, Prentice A, Saridogan E, Soriano D, Nelen W, European Society of Human Reproduction and Embryology (2014). ESHRE guideline: management of women with endometriosis. Hum Reprod.

[12] Falcone T, Lebovic DI (2011). Clinical management of endometriosis. Obstet Gynecol.

[13] Johnson NP, Hummelshoj L, World Endometriosis Society Montpelier Consortium (2013). Consensus on current management of endometriosis. Hum Reprod.

[14] Kuznetsov L, Dworzynski K, Davies M, Overton C, Committee Guideline (2017). Diagnosis and management of endometriosis: summary of NICE guidance. BMJ.

[15] Leyland N, Casper R, Laberge P, Singh SS (2010). Endometosis: diagnosis and management. J Obstet Gynaecol Can.

[16] Practice Committee of the American Society for Reproductive Medicine (2014). Treatment of pelvic pain associated with endometriosis: a committee opinion. Fertil Steril.

[17] Grandi G, Mueller M, Bersinger NA, Cagnacci A, Volpe A, McKinnon B (2016). Does dienogest influence the inflammatory response of endometriotic cells? A systematic review. Inflamm Res.

[18] Miyashita M, Koga K, Takamura M, Izumi G, Nagai M, Harada M, Hirata T, Hirota Y, Fujii T, Osuga Y (2014). Dienogest reduces proliferation, aromatase expression and angiogenesis, and increases apoptosis in human endometriosis. Gynecol Endocrinol.

[19] Nirgianakis K, Grandi G, McKinnon B, Bersinger N, Cagnacci A, Mueller M (2016). Dienogest mediates midkine suppression in endometriosis. Hum Reprod.

[20] Katsuki Y, Takano Y, Futamura Y, Shibutani Y, Aoki D, Udagawa Y, Nozawa S (1998). Effects of dienogest, a synthetic steroid, on experimental endometriosis in rats. Eur J Endocrinol.

[21] Strowitzki T, Faustmann T, Gerlinger C, Seitz C (2010). Dienogest in the treatment of endometriosis-associated pelvic pain: a 12-week, randomized, double-blind, placebo-controlled study. Eur J Obstet Gynecol Reprod Biol.

[22] Strowitzki T, Marr J, Gerlinger C, Faustmann T, Seitz C (2010). Dienogest is as effective as leuprolide acetate in treating the pain- ful symptoms of endometriosis: a 24-week, randomized, multi-center, open-label trial. Hum Reprod.

[23] Strowitzki T, Marr J, Gerlinger C, Faustmann T, Seitz C (2012). Detailed analysis of a randomized, multicenter, comparative trial of dienogest versus leuprolide acetate in endometriosis. Int J Gynaecol Obstet.

[24] Morotti M, Sozzi F, Remorgida V, Venturini PL, Ferrero S (2014). Dienogest in women with persistent endometriosis-related pelvic pain during norethisterone acetate treatment. Eur J Obstet Gynecol Reprod Biol.

[25] Vercellini P, Bracco B, Mosconi P, Roberto A, Alberico D, Dhouha D, Somigliana E (2016). Norethindrone acetate or dienogest for the treatment of symptomatic endometriosis: a before and after study. Fertil Steril.

[26] Momoeda M, Harada T, Terakawa N, Aso T, Fukunaga M, Hagino H, Taketani Y (2009). Long-term use of dienogest for the treatment of endometriosis. J Obstet Gynaecol Res.

[27] Petraglia F, Hornung D, Seitz C, Faustmann T, Gerlinger C, Luisi S, Lazzeri L, Strowitzki T (2012). Reduced pelvic pain in women with endometriosis: efficacy of long-term dienogest treatment. Arch Gynecol Obstet.

[28] Schindler AE (2011). Dienogest in long-term treatment of endometriosis. Int J Womens Health.

[29] Kim SA, Um MJ, Kim HK, Kim SJ, Moon SJ, Jung H (2016). Study of dienogest for dysmenorrhea and pelvic pain associated with endometriosis. Obstet Gynecol Sci.

[30] Leonardo-Pinto JP, Benetti-Pinto CL, Cursino K, Yela DA (2017). Dienogest and deep infiltrating endometriosis: the remission of symptoms is not related to endometriosis nodule remission. Eur J Obstet Gynecol Reprod Biol.

[31] Maiorana A, Incandela D, Parazzini F, Alio W, Mercurio A, Giambanco L, Alio L (2017). Efficacy of dienogest in improving pain in women with endometriosis: a 12-month single-center experience. Arch Gynecol Obstet.

[32] Ota Y, Andou M, Yanai S, Nakajima S, Fukuda M, Takano Mi, Kurotsuchi S, Ebisawa K, Hada T, Ota I (2015). Long-term administration of dienogest reduces recurrence after excision of endometrioma. J Endometr Pelvic Pain Disord..

[33] Yamanaka A, Hada T, Matsumoto T, Kanno K, Shirane A, Yanai S, Nakajima S, Ebisawa K, Ota Y, Andou M (2017). Effect of dienogest on pain and ovarian endometrioma occurrence after laparoscopic resection of uterosacral ligaments with deep infiltrating endometriosis. Eur J Obstet Gynecol Reprod Biol.

[34] Delgado DA, Lambert BS, Boutris N (2018). Validation of digital visual analog scale pain scoring with a traditional paper-based visual analog scale in adults. J Am Acad Orthop Surg Glob Res Rev.

[35] Correll DJ (2007) Chapter 18—the measurement of pain: objectifying the subjective. In: Waldman SD, Bloch JI (eds) Pain management. WB Saunders, pp 197–211. 10.1016/B978-0-7216-0334-6.50022-4 (**ISBN 9780721603346**)

[36] Cho B, Roh JW, Park J, Jeong K, Kim TH, Kim YS, Kwon YS, Cho CH, Park SH, Kim SH (2020). Safety and effectiveness of dienogest (Visanne®) for treatment of endometriosis: a large prospective cohort study. Reprod Sci.

[37] Lee SR, Yi KW, Song JY, Seo SK, Lee DY, Cho S, Kim SH (2018). Efficacy and safety of long-term use of dienogest in women with ovarian endometrioma. Reprod Sci.

[38] Uludag SZ, Demirtas E, Sahin Y, Aygen EM (2021). Dienogest reduces endometrioma volume and en-dometriosis-related pain symptoms. J Obstet Gynaecol.

[39] Angioni S, Pontis A, Malune ME, Cela V, Luisi S, Litta P, Vignali M, Nappi L (2020). Is dienogest the best medical treatment for ovarian endometriomas? Results of a multicentric case control study. Gynecol Endocrinol.

[40] Xholli A, Filip G, Previtera F, Cagnacci A (2020). Modification of endometrioma size during hormone therapy containing dienogest. Gynecol Endocrinol.

[41] Zakhari A, Edwards D, Ryu M, Matelski JJ, Bougie O, Murji A (2020). Dienogest and the risk of endo-metriosis recurrence following surgery: a systematic review and meta-analysis. J Minim Invasive Gynecol.

